# Child Stunting is Associated with Low Circulating Essential Amino Acids

**DOI:** 10.1016/j.ebiom.2016.02.030

**Published:** 2016-02-19

**Authors:** Richard D. Semba, Michelle Shardell, Fayrouz A. Sakr Ashour, Ruin Moaddel, Indi Trehan, Kenneth M. Maleta, M. Isabel Ordiz, Klaus Kraemer, Mohammed A. Khadeer, Luigi Ferrucci, Mark J. Manary

**Affiliations:** a Wilmer Eye Institute, Johns Hopkins University School of Medicine, Baltimore, MD, USA; b National Institute on Aging, National Institutes of Health, Baltimore, MD, USA; c Department of Nutrition & Food Science, College of Agriculture and Natural Resources, University of Maryland, College Park, MD, USA; d Department of Pediatrics, Washington University in St. Louis, St. Louis, MO, USA; e School of Public Health and Family Medicine, University of Malawi College of Medicine, Blantyre, Malawi; f Sight and Life, Basel, Switzerland; g Department of International Health, Johns Hopkins Bloomberg School of Public Health, Baltimore, MD, USA

**Keywords:** Amino acids, Children, Glycerophospholipids, Malnutrition, Sphingolipids, Stunting

## Abstract

**Background:**

Stunting affects about one-quarter of children under five worldwide. The pathogenesis of stunting is poorly understood. Nutritional interventions have had only modest effects in reducing stunting. We hypothesized that insufficiency in essential amino acids may be limiting the linear growth of children.

**Methods:**

We used a targeted metabolomics approach to measure serum amino acids, glycerophospholipids, sphingolipids, and other metabolites using liquid chromatography-tandem mass spectrometry in 313 children, aged 12–59 months, from rural Malawi. Children underwent anthropometry.

**Findings:**

Sixty-two percent of the children were stunted. Children with stunting had lower serum concentrations of all nine essential amino acids (tryptophan, isoleucine, leucine, valine, methionine, threonine, histidine, phenylalanine, lysine) compared with nonstunted children (p < 0.01). In addition, stunted children had significantly lower serum concentrations of conditionally essential amino acids (arginine, glycine, glutamine), non-essential amino acids (asparagine, glutamate, serine), and six different sphingolipids compared with nonstunted children. Stunting was also associated with alterations in serum glycerophospholipid concentrations.

**Interpretation:**

Our findings support the idea that children with a high risk of stunting may not be receiving an adequate dietary intake of essential amino acids and choline, an essential nutrient for the synthesis of sphingolipids and glycerophospholipids.

## Highlights

• We used metabolomics and mass spectrometry to gain insight into nutrition of stunted children.
• Stunted children in rural Africa had low circulating levels of essential amino acids.
• Children at risk of stunting may not have an adequate dietary intake of essential amino acids.

Worldwide, one-quarter of children under five years are short for their age (stunted), indicative of chronic malnutrition. Lipid-based nutrient supplements containing micronutrients have little to no effect in reducing child stunting. We examined the relationship between circulating metabolites with stunting in young children in Africa. Stunted children had lower serum levels of all nine essential amino acids compared with non-stunted children. These results challenge the widespread assumption that protein intake is adequate among young children in developing countries. The findings support the idea that children at high risk of stunting are not receiving adequate dietary intake of essential amino acids.

## 1 Introduction

Stunting affects about one-quarter of children under five years of age worldwide (Black et al., 2013, UNICEF/World Health Organization/World Bank Group, 2015). Stunting is considered the best available summary measure of chronic malnutrition. Child stunting may develop during the first two years of life and is largely attributed to inadequate nutrition and infectious diseases (Black et al., 2013). In 2014, there were an estimated 159 million children who were stunted, with nearly all stunted children living in low-income countries (UNICEF/World Health Organization/World Bank Group, 2015). Stunting is associated with decreased survival, impaired cognitive and motor development, reduced economic productivity, and higher chance of living in poverty in adulthood (Black et al., 2013, Grantham-McGregor et al., 2007). The World Health Assembly has set a global target of a 40% reduction in the number of stunted under-five children by 2025 (de Onis et al., 2013). This targeted reduction in child stunting is included in the United Nations Sustainable Development Goal #2 (Murray, 2015). Nutritional interventions only have a modest impact upon stunting and will be insufficient to meet this goal alone. Even if ten evidence-based nutritional interventions were all applied at 90% coverage, stunting would be reduced by only ~ 20% (Bhutta et al., 2013), which falls short of international goals to reduce stunting. The pathogenesis of stunting remains poorly understood. There may be as yet unknown or limiting nutritional factors that contribute to child stunting.

In the 1950s and 1960s, international organizations were focused on protein malnutrition in children in developing countries (Semba, 2008). The emphasis shifted away from proteins to micronutrient malnutrition in the 1970s (Semba, 2008), under the assumption that most children received adequate protein. This assumption needs to be re-examined for several reasons. New evidence shows human growth is controlled by the master growth regulation pathway, the mechanistic target of rapamycin complex C1 (mTORC1) (Laplante and Sabatini, 2012). When specific amino acids are deficient in the diet, mTORC1 senses amino acid deficiency and represses protein and lipid synthesis and cellular growth (Laplante and Sabatini, 2012). The linear growth of children is dependent upon the chondral growth plate (Baron et al., 2015). Bone growth by the chondral plate is regulated by mTORC1 and the availability of amino acids, such as the essential amino acid leucine (Kim et al., 2009). Children at high risk of stunting may have limitations of essential amino acids in their diet such as tryptophan and lysine (Nuss and Tanumihardjo, 2011). The amino acid requirements of young children were not directly established and are currently derived based on a factorial computation (Pillai and Kurpad, 2012). Whether current dietary recommendations of essential amino acids are sufficient for children in low-income settings – where infectious diseases are common and catch-up growth is important – is unclear. The burden of infectious disease and metabolic needs for immune system activation may partition limited essential amino acids to support immune function at the expense of growth (Kampman-van de Hoek et al., 2016).

Recent advances in metabolomics and mass spectrometry now facilitate rapid and absolute quantification of serum amino acids and other metabolites in large epidemiological studies. We hypothesized that stunted children have decreased concentrations of circulating essential amino acids and other metabolites. To address this hypothesis, we used a targeted metabolomics approach to investigate serum amino acids and other metabolites in a cohort of young children in rural Malawi.

## 2 Methods

### 2.1 Study Design and Participants

The study design was cross-sectional. The study subjects consisted of a community-based sample of 313 children, aged 12–59 months, seen in six villages (Masika, Makhwira, Mitondo, Mbiza, Chamba, and Mayaka) in rural southern Malawi in 2011. Children were eligible for the study if they had no evidence of severe acute malnutrition, congenital or chronic disease, or caretaker-reported diarrhea. The study enrolled 540 children, of whom venous blood samples were obtained from 483 children. A simple random sample of 313 children was used for the analysis of serum metabolites. Children underwent anthropometry conducted by trained, experienced field workers. Weight was measured to the nearest 5 g using a digital scale (Seca 334, Chino, CA). Recumbent length (for children < 24 months) or standing height was measured to the nearest 0.1 cm using a rigid height board (Seca 417). Height-for-age Z-scores (HAZ) and weight-for-height Z-scores were calculated using World Health Organization growth curves (de Onis et al., 2012). Stunting was defined as HAZ < − 2 (de Onis et al., 2012). Chichewa-speaking Malawian research nurses obtained written and oral informed consent from each child's caretaker before enrollment in the study. Community consent for the study also was obtained from the village chief and local health officials. The protocol for this study was approved by the College of Medicine Research Ethics Committee of the University of Malawi, the Human Research Protection Office of Washington University in St. Louis, and the Johns Hopkins School of Medicine Institutional Review Board.

### 2.2 Measurement of Serum Metabolites

Venous blood was drawn by study nurses and doctors. Serum samples were processed and snap frozen in liquid nitrogen within 4 h of blood drawing. Cryovials were stored at − 80 °C. Sera were not thawed prior to metabolite measurements. Samples with no hemolysis were used for the analyses. Serum metabolites were measured in a masked fashion using liquid chromatography tandem mass spectrometry (LC-MS/MS). Metabolites were extracted and concentrations measured using the AbsoluteIDQ p180 kit (Biocrates Life Sciences AG, Innsbruck, Austria) following the manufacturers protocol for a 5500 QTrap (Sciex, Framingham, MA) mass spectrometer equipped with an electrospray ionization source, a CBM-20A command module, LC-20AB pump, and a SIL-20AC-HT autosampler, a CTO-10Ac column oven heater (all Shimadzu, Tokyo, Japan), and running with Analyst 1.5.2 software (Biocrates), as described in detail elsewhere (Schmerler et al., 2012). The method measured 139 metabolites: 22 amino acids, 3 biogenic amines, 6 amino acid metabolites, 15 sphingolipids, 8 acylcarnitines, and 85 glycerophospholipids (lyso-, diacyl-, and acyl-alkyl phosphatidylcholines). Glycerophospholipids are differentiated on the basis of ester and ether bonds in the glycerol moiety. Diacyl or “aa” indicates that fatty acids are bound with ester bonds at the sn-1 and sn-2 positions on the glycerol backbone. Acyl-alkyl or “ae” indicates that the fatty acid at the sn-1 position is bound with an ether bond. The total number of carbon atoms and double bonds in fatty acid chains is represented by “C x:y”, where x denotes the number of carbons and y denotes the number of double bonds. Phosphatidylcholine (PC), lysophosphatidylcholine (lysoPC), and sphingomyelin (SM), and hydroxysphingomyelin (SM [OH]) are used in the abbreviations. The MS spectra were evaluated using Analyst/MetIDQ software (Biocrates). Human serum samples spiked with standard metabolites were used to monitor the reproducibility of the assay. Metabolites that were below the limit of quantification were excluded. The inter-assay and intra-assay coefficients of variation ranged from 5% to 15% for nearly all analytes.

### 2.3 Statistical Analysis

The sample size of 313 children was based upon > 90% power to detect at least a 10% difference in serum leucine between stunted and non-stunted children, given a 60% prevalence of stunting, σ = 47 μmol/L (Reinehr et al., 2015), no matching, α = 0.05, and a two-sided test. Of all the essential amino acids, we chose leucine for power calculations, since it is the most well-characterized amino acid sensed by the mTORC1 pathway (Laplante and Sabatini, 2012, Saxton et al., 2016). Univariate exploratory data analyses using histograms and boxplots were used to examine the distribution of serum metabolites. Linear regression was performed of HAZ on serum metabolites in separate models using one model per metabolite and a combined model with all metabolites. Bivariate exploratory analyses were used to relate each metabolite to HAZ and to each other to ensure linear relations between metabolites and HAZ. Spearman correlations were used to examine correlations between HAZ and serum metabolites. Wilcoxon rank-sum test, adjusted by age and gender, was used to compare serum metabolites between stunted and non-stunted children. A Bonferroni adjustment to type I error was made to account for the multiple metabolites, many which are closely correlated, into five general classes (amino acids, biogenic amines/amino acid metabolites, sphingolipids, acylcarnitines, glycerophospholipids) so that p-value < 0.01 (= 0.05/5) was considered statistically significant. Statistical analyses were conducted using R version 3.1.

### 2.4 Role of the Funding Source

The study sponsors had no role in the study, writing of the report, or decision to submit the paper.

## 3 Results

**Fig. 1:**
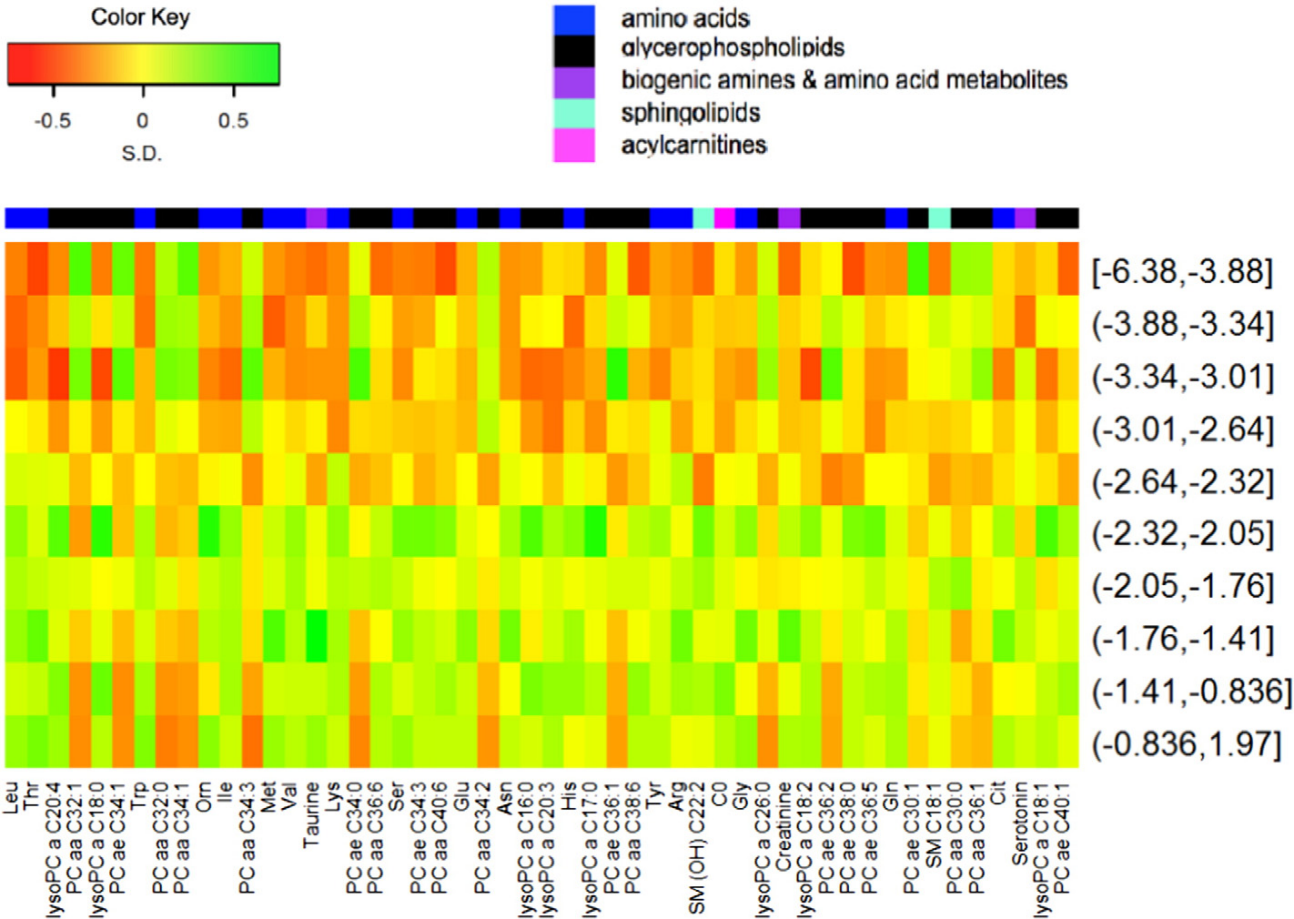
Heat map showing the relationship of the fifty most significant serum metabolites by HAZ, adjusted by age and gender. HAZ divided into deciles. Metabolites are ordered by p-values, with the lowest p-values on the left side. Standard three-letter abbreviations used for amino acids. Abbreviations for lipid nomenclature are described in the methods section. Other abbreviation: carnitine (C0).

The characteristics of the 313 children in the study are shown in Table 1. There were nearly equal numbers of girls and boys. Over 60% (194/313) of the children were stunted. A summary heatmap of the top fifty serum metabolites by HAZ is shown in Fig. 1. Serum amino acid and biogenic amines in children with and without stunting, adjusted by age and gender, are shown in Supplementary Table 1. All nine essential amino acids (tryptophan, isoleucine, leucine, valine, methionine, threonine, histidine, phenylalanine, lysine), three conditionally essential amino acids (arginine, glycine, glutamine), three non-essential amino acids (asparagine, glutamate, serine), and citrulline were significantly lower in stunted compared with non-stunted children. There were no significant differences in serum proline, tyrosine, alanine, and aspartic acid between children with and without stunting. Of the biogenic amines and amino acid metabolites, serum ornithine, taurine, and asymmetric dimethylarginine were significantly lower in stunted compared with non-stunted children. There were no significant differences in serum alpha-aminoadipic acid, kynurenine, creatinine, spermine, putrescine, symmetric dimethylarginine, and total dimethylarginine between stunted and non-stunted children.

**Table 1:** Characteristics of the study population.

Characteristic	Mean (SD) or %
Age, months	34.6 (11.8)
Female, %	49.7
Weight-for-height Z-score	0.2 (0.9)
Height-for-age Z-score	− 2.3 (1.3)
Stunted,^a^ %	62
Caretaker is mother, %	95
Father is alive, %	95
Siblings, n	3.5 (1.6)
Individuals that sleep in same room as child, n	3.3 (1.5)
Home with a metal roof, %	20
Family owns bicycle, %	61
Animals sleep in house, %	37
Water from a clean source, %	70
Child uses pit latrine, %	79

a Height-for-age Z-score < − 2.

Serum sphingolipid and acylcarnitine concentrations in children with and without stunting, adjusted by age and gender, are shown in Supplementary Table 2. Three hydroxysphingomyelins (SM [OH] C14:1, CM [OH] C16:1, SM [OH] C22:2), three sphingomyelins (SM C16:1, SM C18:0, SM C18:1), and carnitine were significantly lower in stunted compared with non-stunted children. A detailed heatmap showing the relationship of serum amino acids, biogenic amines, amino acid metabolites, acylcarnitines, and sphingolipids by HAZ is shown in Supplementary Fig. 1.

Serum glycerophospholipid concentrations in children with and without stunting, adjusted by age and gender, are shown in Supplementary Table 3. Seven lysophosphatidylcholines (lysoPC a C16:0, lysoPC a C17:0, lysoPC a C18:0, lysoPC a C18:1, lysoPC a C18:2, lysoPC a C20:3, lysoPC a C20:4), four diacyl-phosphatidylcholines (PC aa C28:1, PC aa C34:4, PC aa C36:6, PC aa C38:6), and three acyl-alkyl-phosphatidylcholines (PC ae C34:3, PC ae C36:5, PC ae C38:6) were significantly lower and one lysophosphatidycholine (lyso PC a C26:0), six diacyl-phosphatidylcholines (PC aa C32:0, PC aa C32:1, PC aa C34:1, PC aa C34:2, PC aa C34:3, PC aa C36:1,) and seven acyl-alkyl-phosphatidylcholines (PC ae C34:0, PC ae C34:1, PC ae C36:1, PC ae C36:2, PC ae C38:1, PC ae C38:2, PC ae C42:1) were significantly higher in stunted compared with non-stunted children. A detailed heatmap showing the relationship of serum glycerophospholipids by HAZ is shown in Supplementary Fig. 2.

**Fig. 2:**
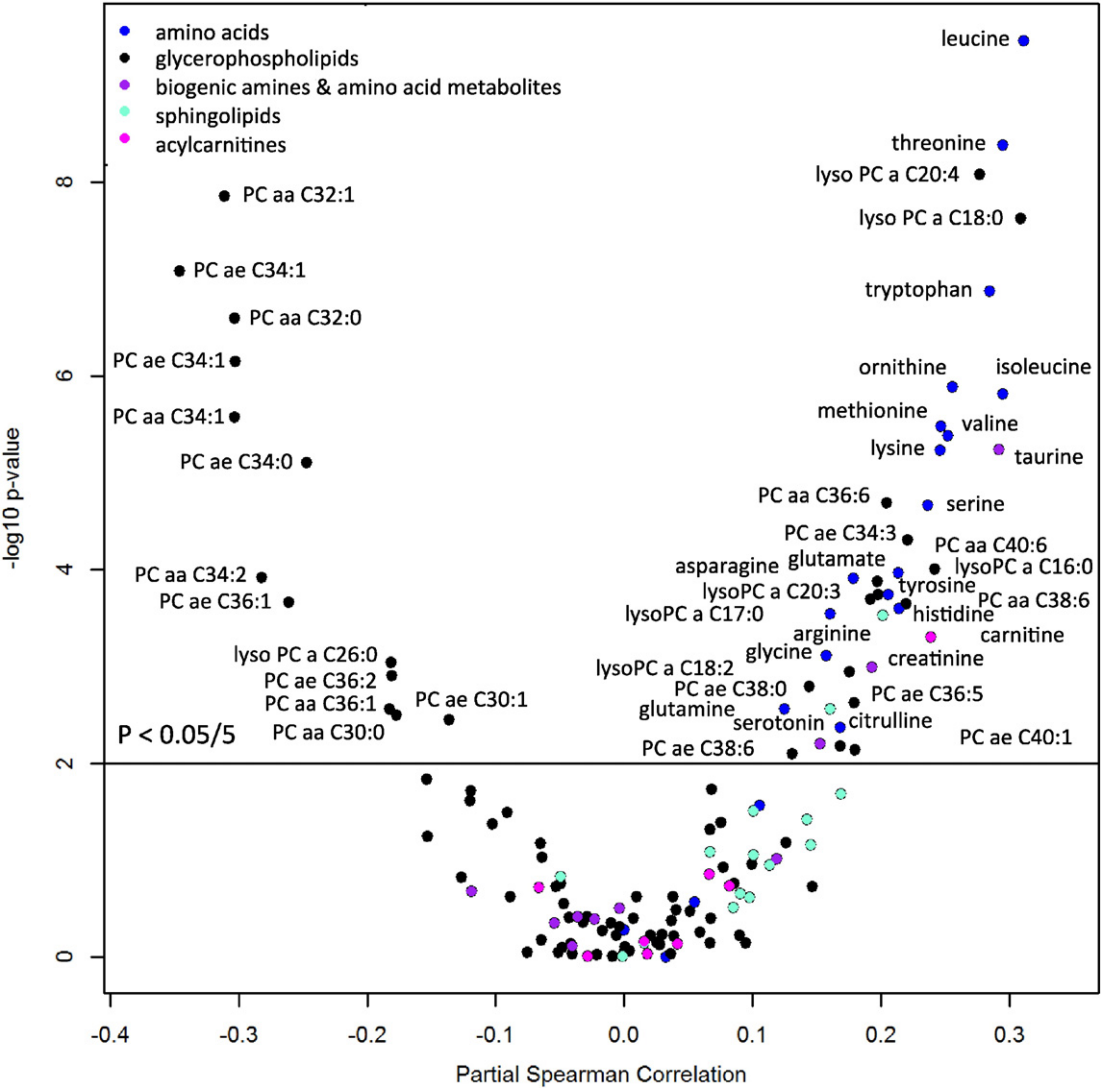
Volcano plot showing the relationship of partial Spearman correlations, adjusted for age and gender, between HAZ and serum metabolites using Bonferroni-adjusted p-values. Horizontal line indicates p-value cut-off for Bonferroni-adjusted p-value of 0.05/5 (p < 0.01).

The relationship of HAZ with all serum metabolites is summarized in a volcano plot in Fig. 2. Of the 19 proteinogenic amino acids measured (cysteine not measured in this assay), 16 amino acids had a significant positive correlation with HAZ, including eight of nine essential amino acids (tryptophan, isoleucine, leucine, valine, methionine, threonine, histidine, lysine), four conditionally essential amino acids (arginine, glycine, glutamine, tyrosine), and three non-essential amino acids (asparagine, glutamate, serine). Non-proteinogenic amino acids (citrulline, ornithine, taurine) and one amino acid metabolite (creatinine) had a significant positive association with HAZ. One biogenic amine (serotonin) had a significant positive association with HAZ. Serum phenylalanine, proline, alanine, aspartic acid, alpha-aminoadipic acid, kynurenine, spermine, putrescine, total dimethylarginine, symmetric dimethylarginine, and asymmetric dimethylarginine were not significantly associated with HAZ.

Two serum sphingomyelins (SM [OH] C22:2, SM C18:0) and serum carnitine had significant positive correlations with HAZ. Seven serum lysophosphatidylcholines (lysoPC a C16:0, lysoPC a C17:0, lysoPC a C18:0, lysoPC a C18:1, lysoPC a C18:2, lysoPC a C20:3, lyso PC a C20:4), three serum diacyl-phosphatidylcholines (PC aa C36:6, PC aa C38:6, PC aa C40:6), and five acyl-alkyl-phosphatidylcholines (PC ae C34:3, PC ae C36:5, PC ae C38:0, PC ae C38:6, PC ae C40:1) had significant positive correlations with HAZ. One lysophosphatidylcholine (lyso PC a C26:0), seven serum diacyl-phosphatidylcholines (PC aa C30:0, PC aa C32:0, PC aa C32:1, PC aa C34:1, PC aa C34:2, PC aa C34:3, PC aa C36:1), and six acyl-alkyl-phosphatidylcholines (PC ae C30:1, PC ae C34:0, PC ae C34:1, PC ae C36:1, PC ae C36:2, PC ae C38:2) had significant negative correlations with HAZ.

**Fig. 3:**
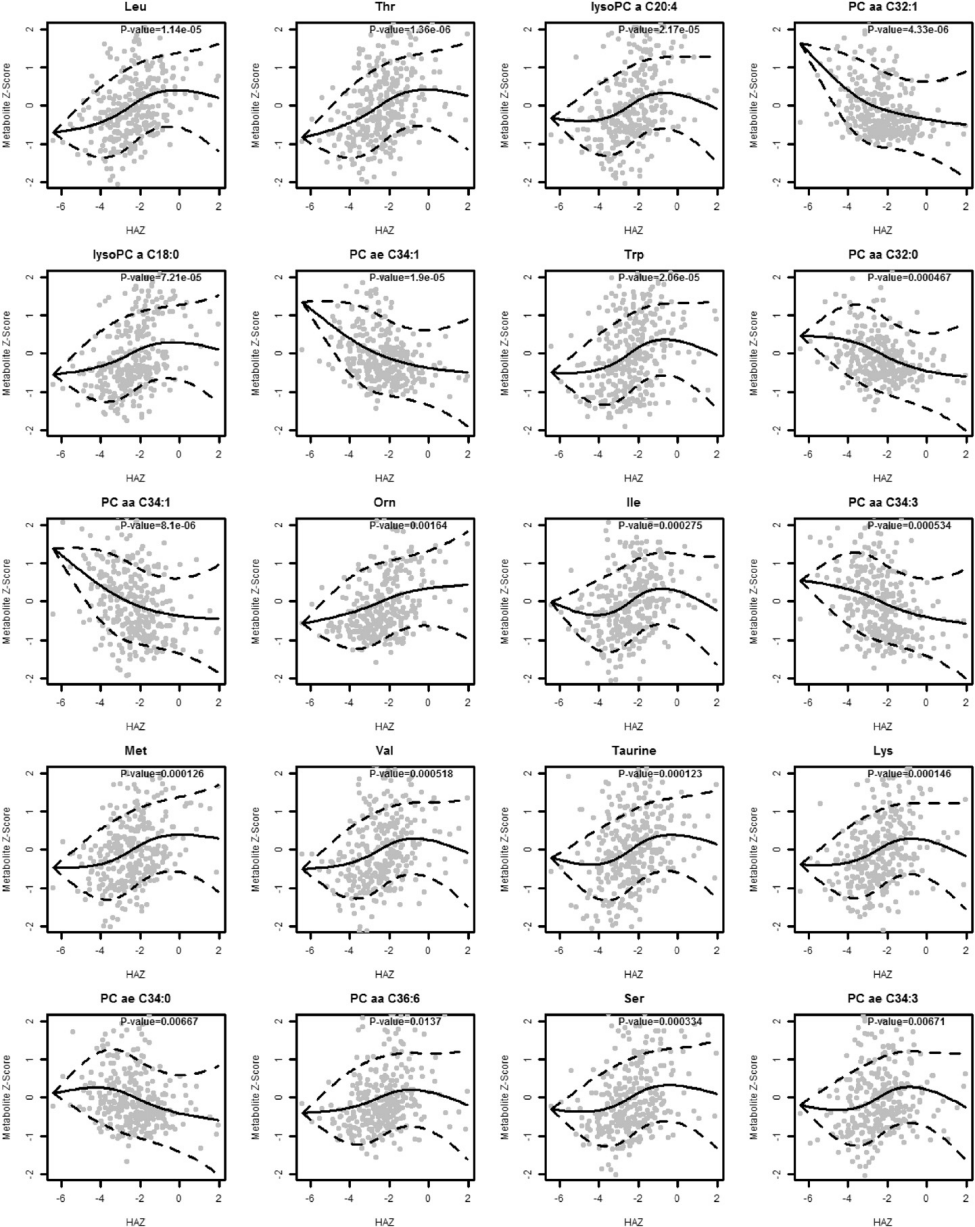
Scatterplots and fitted regression curves using natural cubic splines for the 20 serum metabolites with the most significant correlations. Abbreviations for lipid nomenclature are described in the methods section. Standard three-letter abbreviations used for amino acids.

The Spearman correlations between HAZ and serum amino acids and biogenic amines, serum sphingolipids and acylcarnitines, and serum glycerophospholipids are shown in Supplementary Tables 4–6. Scatterplots and fitted regression curves using natural cubic splines are shown for the 20 most significant metabolites in Fig. 3.

## 4 Discussion

The present study shows that stunted children have lower serum concentrations of all nine essential amino acids. In addition, stunting was associated with lower circulating levels of three conditionally essential amino acids, other proteinogenic amino acids, biogenic amines, amino acid metabolites, sphingomyelins, and with alterations in glycerophospholipids. As noted, in response to low amino acid availability, mTORC1 represses synthesis of both proteins and lipids, thus limiting cell growth (Laplante and Sabatini, 2012). Another amino acid sensing and growth pathway related to mTORC1 is the general control nonderepressible 2 (GCN2) pathway (Efeyan et al., 2015, Ye et al., 2015). The changes in serum amino acids in the fasting versus non-fasting states can be detected by nutrient sensing pathways mTORC1 and GCN2 (Efeyan et al., 2015, Laplante and Sabatini, 2012). Thus, the low serum amino acid concentrations found in these children have the potential to repress protein and lipid synthesis through mTORC1 and GCN2 and retard growth. Leucine, a key amino acid sensed by mTORC1 (Efeyan et al., 2015, Laplante and Sabatini, 2012, Saxton et al., 2016), was the metabolite with the strongest association with linear growth in the present study.

Essential amino acids are considered “essential” because they cannot be synthesized by the body and must be obtained from diet (Wu, 2009). Insufficient intake of essential amino acids could adversely affect multiple metabolic pathways since they play diverse roles in human health. Methionine is a precursor to homocysteine, cysteine, and taurine, and to *S*-adenosylmethionine, the primary methyl donor in the synthesis of polyamines (Wu, 2009). Tryptophan is the precursor for niacin and for serotonin, a neuromediator primarily found in the enterochromaffin cells of the gut (Wu, 2009). Lysine is the precursor to carnitine and is required for the structural modification of collagens (Wu, 2009). Threonine is a major component of secretory mucin 2 that forms the protective mucus layer of the gut (Wu, 2009). Histidine plays a role in protein methylation, hemoglobin structure and function, and is a precursor for both histamine and carnosine (Wu, 2009). Phenylalanine is a precursor for tyrosine, the substrate for synthesis of catecholamines (Wu, 2009).

Restriction of conditionally essential amino acids in the diet could also impact health. Arginine serves as a precursor for the synthesis of nitric oxide, creatine, symmetric and asymmetric dimethylarginine, and polyamines, and is interconvertible with proline and glutamate (Wu, 2009). Glutamine is essential to enterocyte growth and intestinal barrier function (Wu, 2009). Glycine plays a role in protein synthesis, synthesis of purines, conjugation of bile acids, as a neurotransmitter in the central nervous system, and in cytoprotection (Wu, 2009).

In the present study, stunted children had lower serum sphingomyelins. Sphingomyelins are dominant sphingolipids in membranes of mammalian cells and are mainly localized to the plasma membrane, especially the outer leaflet, and to the endocytic recycling compartment and the trans-Golgi network (Slotte, 2013). Sphingomyelins play a central role in creating lateral structures in membranes for Toll-like receptors, class A and B scavenger receptors, and insulin receptors (Slotte, 2013). Sphingomyelins are involved in cell signaling (Wu, 2009), and T cell activation and differentiation (Beyersdorf and Müller, 2015). Sphingomyelins are a major lipid component of myelin and are essential to myelination of the central nervous system during child development. Myelination of the central nervous system is dependent upon the mTORC1 pathway, and activation of mTORC1 for myelination requires amino acid sufficiency (Lebrun-Julien et al., 2014). The synthesis of sphingomyelins is closely related with phosphatidylcholines, since the final step in biosynthesis of sphingomyelins is dependent upon phosphatidylcholines, the donor of a phosphorylcholine group to ceramide.

In the present study, fourteen phosphatidylcholines were significantly lower and fourteen were significantly higher in children with stunting, which is suggestive of wide alterations in phosphatidylcholine synthesis or catabolism. Phosphatidylcholines are the most abundant phospholipid in mammalian cell membranes and the dominant phospholipid circulating in plasma (Fagone and Jackowski, 2013). Cell proliferation, differentiation, and growth are highly dependent upon phosphatidylcholines, since they serve as the major component of cell membranes (Fagone and Jackowski, 2013). Phosphatidylcholines are essential for lipoprotein assembly and secretion by the liver (Cole et al., 2012), and comprise the main active component of lung surfactant. Phosphatidylcholines and lyso-phosphatidylcholines, which are derived from phosphatidylcholines, account for > 90% of the lipid in the protective mucus layer in the gut (Johansson et al., 2011). Both phosphatidylcholines and sphingomyelins are involved in chondrogenesis, a major determinant of linear growth (Baron et al., 2015). Endochondral bone formation, which plays a key role in linear growth of bones, requires synthesis of phosphatidylcholines (Kular et al., 2015, Li et al., 2014).

Phosphatidylcholines are mainly synthesized in the CDP-choline pathway that begins with the absorption of dietary choline in the small intestine and involves three enzymatic steps. The second pathway for phosphatidylcholine synthesis is the phosphatidylethanolamine N-methyltransferase (PEMT) pathway, which is quantitatively significant only in liver and not other tissues. Choline is an essential nutrient since the PEMT pathway is insufficient to supply the body's need for choline (Food and Nutrition Board, Institute of Medicine, 1998). Low serum phosphatidylcholine concentrations are potentially correctable with increased intake of choline-rich foods. Children with stunting may be at greater risk of choline deficiency due to a choline-poor diet. The richest dietary sources of choline are mostly animal-based foods (Zeisel and da Costa, 2009), which are not regularly consumed by poor families in low-income countries. A recent study of urinary metabolites suggests that biochemical pathways involving choline and tryptophan metabolism are associated with catch-up growth in undernourished Brazilian children (Mayneris-Perxaches et al., 2016).

The findings from this study cannot necessarily be extrapolated to other children at risk of stunting, since there may be dietary, cultural, and environmental factors that differ from the setting in rural Malawi. Serum metabolite concentrations may change over time. However, studies in adults show that the intra-class correlation coefficients for repeated measurements of the metabolites involved in this study are moderately high (Breier et al., 2014). Although the mTORC1 and GCN2 pathways provide a biological framework for interpreting the findings of this study, the present study is limited in that it provides no direct evidence of alterations in the mTORC1 or GCN2 pathways. Such studies could be undertaken in the future and would likely require evidence on the level of the transcriptome or proteome.

The strengths of this study are the community-based sample of children in rural Africa in a setting where stunting is common, the use of gold standard LC–MS/MS methodology, and a well-characterized and validated platform for absolute quantification of serum metabolites (Breier et al., 2014, Reinehr et al., 2015, Schmerler et al., 2012). There is currently little reference data available on serum amino acid concentrations in healthy children where LC–MS/MS has been used for absolute quantification of amino acids.

This study suggests that the dietary intake of essential amino acids may be insufficient in children with stunting. An insufficiency in essential amino acids could potentially explain why micronutrient and lipid supplements have had little to no effect on child growth (Ashorn et al., 2015, Garza, 2015, Mayo-Wilson et al., 2014, Ramakrishnan et al., 2009, Stammers et al., 2015, van der Merwe et al., 2013). mTORC1 and GCN2 growth regulatory pathways will not allow growth to proceed if amino acids are not sufficient to synthesize proteins (Efeyan et al., 2015, Laplante and Sabatini, 2012). The findings of the study also support the notion that stunted children do not receive sufficient dietary choline, as reflected by low serum sphingomyelins and alterations in phosphatidylcholines. Future studies are needed to characterize serum choline in children with stunting. The present study is cross-sectional, thus, causality cannot be necessarily inferred between essential amino acids and stunting. Longitudinal prospective assessments of serum amino acids and other metabolites will be critical in understanding the association with linear growth in children. Corroboration from longitudinal studies would provide the needed rationale for randomized clinical trials. Randomized controlled trials would ultimately be required to determine whether essential amino acids and choline play a causal role in the pathogenesis of child stunting.

## Contributors

RDS, MS, IT, KK, LF, and MJM were responsible for the study design, statistical analyses, data interpretation, and writing the manuscript. MS and FASA conducted the data analysis. IT, KMM, and MJM implemented the field study in rural Malawi. RM, MAK, and MIO contributed towards laboratory analyses of serum metabolites. All authors contributed to writing and approving the final manuscript. The corresponding author had full access to all the data in the study and had final responsibility for the decision to submit for publication.

## Declaration of Interests

The authors declare no competing financial interests.
