# Supplementary material for: Child Stunting is Associated with Low Circulating Essential Amino Acids
Source: eBioMedicine. 2016 Feb 19;6:246–52. doi: 10.1016/j.ebiom.2016.02.030 (PMC4856740; doi:10.1016/j.ebiom.2016.02.030)
Supplement: Supplementary file 1 — Supplementary materials [file mmc1.docx]

**ONLINE SUPPLEMENTARY INFORMATION**

**Supplementary Table 1. Serum amino acid, biogenic amine, and amino acid metabolite concentrations in children with and without stunting, adjusted by age and gender**

| **Analyte in μmol/L** | **Stunted**  **(n = 194)** | | **Not Stunted**  **(n = 119)** | | ***P*^1^** | **Analyte in μmol/L** | **Stunted**  **(n = 194)** | | **Not Stunted**  **(n = 119)** | | ***P*^1^** |
| --- | --- | --- | --- | --- | --- | --- | --- | --- | --- | --- | --- |
|  | **Median** | **IQR** | **Median** | **IQR** |  |  | **Median** | **IQR** | **Median** | **IQR** |  |
| Tryptophan | 32.6 | 21.3, 46.1 | 44.8 | 34.0, 57.0 | <0.0001* | Alanine | 481.6 | 397.6, 575.7 | 497.7 | 440.0, 584.9 | 0.08 |
| Isoleucine | 53.6 | 44.7, 62.1 | 62.9 | 53.6, 72.7 | <0.0001* | Aspartic acid | 53.7 | 40.7, 69.9 | 58.9 | 47.2, 70.5 | 0.19 |
| Leucine | 126.9 | 102.7, 150.8 | 152.0 | 128.5, 172.7 | <0.0001* | Glutamate | 186.8 | 143.0, 268.9 | 295.8 | 193.7, 363.8 | <0.0001* |
| Valine | 133.5 | 115.4, 164.5 | 157.9 | 137.4, 181.5 | <0.0001* | Citrulline | 19.4 | 15.5, 25.9 | 22.0 | 17.6, 29.1 | 0.003* |
| Methionine | 21.6 | 17.2, 26.0 | 24.7 | 20.7, 30.7 | <0.0001* | Ornithine | 64.9 | 50.3, 92.0 | 91.7 | 66.4, 135.0 | <0.0001* |
| Histidine | 84.2 | 70.4, 103.1 | 99.0 | 83.1, 116.3 | <0.0001* | Alpha-aminoadipic acid | 1.11 | 0.72, 9.91 | 1.06 | 0.71, 1.54 | 0.26 |
| Phenylalanine | 83.4 | 72.5, 96.7 | 88.5 | 79.0, 100.8 | 0.01* | Kynurenine | 2.69 | 1.98, 3.40 | 2.85 | 2.19, 3.53 | 0.40 |
| Threonine | 82.2 | 66.0, 104.9 | 104.4 | 83.7, 124.9 | <0.0001* | Creatinine | 21.2 | 17.1, 25.2 | 21.6 | 18.9, 25.9 | 0.12 |
| Lysine | 127.5 | 102.1, 158.6 | 163.8 | 133.1, 202.5 | <0.0001* | Spermine | 0.08 | 0.06, 0.14 | 0.08 | 0.05, 0.13 | 0.54 |
| Asparagine | 53.7 | 40.7, 69.9 | 58.9 | 47.2, 70.5 | <0.0001* | Putrescine | 0.12 | 0.06, 0.18 | 0.13 | 0.07, 0.21 | 0.22 |
| Glutamine | 523.4 | 432.4, 618.4 | 555.3 | 482.2, 659.4 | 0.009* | Serotonin | 1.29 | 0.75, 1.83 | 1.54 | 1.04, 2.13 | 0.002* |
| Arginine | 79.9 | 67.0, 92.4 | 89.9 | 75.0, 106.1 | 0.0003* | Taurine | 164.7 | 128.5, 197.1 | 197.8 | 167.6, 234.5 | <0.0001* |
| Glycine | 307.2 | 246.6, 368.9 | 339.7 | 283.7, 402.3 | 0.0004* | Total dimethylarginine | 1.22 | 0.89, 1.47 | 1.20 | 0.90, 1.59 | 0.59 |
| Proline | 189.8 | 162.7, 230.0 | 190.5 | 159.1, 223.4 | 0.85 | Symmetric dimethylarginine | 0.46 | 0.14, 0.70 | 0.42 | 0.03, 0.75 | 0.75 |
| Serine | 150.6 | 128.2, 178.8 | 175.3 | 147.1, 232.4 | <0.0001* | Asymmetric dimethylarginine | 0.87 | 0.72, 1.06 | 0.99 | 0.79, 1.54 | 0.0007* |
| Tyrosine | 58.3 | 49.4, 71.3 | 63.0 | 54.3, 75.2 | 0.015 |  | | | | | |

^1^Wilcoxon rank-sum test. IQR, interquartile range.

*Significant at Bonferroni-adjusted p-value of <0.01

**Supplementary Table 2. Serum sphingolipid^1^ and acylcarnitine concentrations in children with and without stunting, adjusted by age and gender**

| **Analyte in μmol/L** | **Stunted**  **(n = 194)** | | **Not Stunted**  **(n = 119)** | | ***P*^2^** | **Analyte in μmol/L** | **Stunted**  **(n = 194)** | | **Not Stunted**  **(n = 119)** | | ***P*^2^** |
| --- | --- | --- | --- | --- | --- | --- | --- | --- | --- | --- | --- |
|  | **Mean** | **SD** | **Median** | **IQR** |  |  | **Mean** | **IQR** | **Median** | **IQR** |  |
| SM (OH) C14:1 | 2.22 | 1.84, 2.67 | 2.43 | 2.08, 2.82 | 0.004* | SM C24:1 | 38.5 | 31.5, 43.6 | 39.8 | 35.4, 45.1 | 0.03 |
| SM (OH) C16:1 | 1.97 | 1.38, 1.88 | 1.66 | 1.42, 1.95 | 0.009* | SM C26:0 | 0.20 | 0.16, 0.24 | 0.19 | 0.17, 0.23 | 0.73 |
| SM (OH) C22:1 | 7.22 | 6.22, 8.69 | 7.75 | 6.25, 8.87 | 0.14 | SM C26:1 | 0.26 | 0.21, 0.32 | 0.28 | 0.23, 0.31 | 0.18 |
| SM (OH) C22:2 | 4.47 | 3.76, 5.26 | 4.97 | 4.32, 5.52 | 0.001* | Carnitine (C0) | 20.7 | 17.9, 25.4 | 25.4 | 20.6, 31.6 | <0.0001* |
| SM (OH) C24:1 | 0.85 | 0.70, 1.03 | 0.86 | 0.74, 1.04 | 0.26 | Acetylcarnitine (C2) | 3.46 | 2.64, 4.94 | 4.76 | 3.29, 7.84 | 0.22 |
| SM C16:0 | 87.8 | 75.6, 104.4 | 92.7 | 82.1, 112.3 | 0.02 | Proprionylcarnitine (C3) | 0.48 | 0.38, 0.64 | 0.49 | 0.38, 0.64 | 0.81 |
| SM C16:1 | 8.37 | 6.94, 10.29 | 9.17 | 8.05, 10.71 | 0.002* | Butyrylcarnitine (C4) | 0.27 | 0.17, 0.34 | 0.30 | 0.19, 0.35 | 0.16 |
| SM C18:0 | 17.2 | 13.4, 21.1 | 19.7 | 15.7, 23.5 | 0.0007* | Hydroxybutyrylcarnitine  (C4-OH [C3-DC]) | 0.36 | 0.26, 0.47 | 0.39 | 0.31, 0.50 | 0.04 |
| SM C18:1 | 5.99 | 4.86, 7.25 | 6.81 | 5.83, 7.76 | 0.0002* | Hexadecanoylcarnitine (C16) | 0.20 | 0.15, 0.25 | 0.21 | 0.17, 0.27 | 0.08 |
| SM C20:2 | 0.57 | 0.45, 0.67 | 0.52 | 0.45, 0.67 | 0.34 | Octadecenoylcarnitine (C18:1) | 0.12 | 0.10, 0.16 | 0.15 | 0.11, 0.18 | 0.16 |
| SM C22:3 | 5.59 | 4.32, 6.79 | 5.69 | 4.64, 7.02 | 0.27 | Octadecadienylcarnitine (C18:1) | 0.10 | 0.08, 0.13 | 0.11 | 0.09, 0.13 | 0.11 |
| SM C24:0 | 20.3 | 17.3, 23.8 | 21.0 | 18.4, 24.2 | 0.14 |  | | | | | |

^1^Abbreviations for lipid nomenclature are described in the Methods section.

^2^Wilcoxon rank-sum test. IQR, interquartile range.

*Significant at Bonferroni-adjusted p-value of <0.01

**Supplementary Table 3. Serum glycerophospholipid^1^ concentrations in children with and without stunting, adjusted by age and gender**

| **Analyte in μmol/L** | **Stunted**  **(n = 194)** | | **Not Stunted**  **(n = 119)** | | ***P*^2^** | **Analyte in μmol/L** | **Stunted**  **(n = 194)** | | **Not Stunted**  **(n = 119)** | | ***P*^2^** |
| --- | --- | --- | --- | --- | --- | --- | --- | --- | --- | --- | --- |
|  | **Median** | **IQR** | **Median** | **IQR** |  |  | **Mean** | **IQR** | **Median** | **IQR** |  |
| LysoPC a C16:0 | 132.0 | 100.1, 184.8 | 195.6 | 132.0, 248.0 | <0.0001* | PC aa C42:0 | 0.66 | 0.55, 0.78 | 0.65 | 0.54, 0.79 | 0.96 |
| LysoPC a C16:1 | 2.10 | 2.23, 4.29 | 3.31 | 2.52, 4.19 | 0.15 | PC aa C42:1 | 0.39 | 0.31, 0.46 | 0.39 | 0.29, 0.49 | 0.73 |
| LysoPC a C17:0 | 2.11 | 1.54, 2.94 | 2.94 | 2.10, 3.97 | <0.0001* | PC aa C42:2 | 0.24 | 0.19, 0.28 | 0.23 | 0.16, 0.28 | 0.16 |
| LysoPC a C18:0 | 41.6 | 31.2, 55.2 | 63.7 | 43.6, 84.9 | <0.0001* | PC aa C42:4 | 0.23 | 0.19, 0.30 | 0.22 | 0.19, 0.27 | 0.12 |
| LysoPC a C18:1 | 19.7 | 14.7, 25.3 | 23.0 | 18.8, 26.4 | 0.0003* | PC aa C42:5 | 0.42 | 0.34, 0.51 | 0.40 | 0.33, 0.48 | 0.11 |
| LysoPC a C18:2 | 20.8 | 15.7, 25.9 | 23.6 | 19.3, 30.2 | 0.0003* | PC aa C42:6 | 0.50 | 0.42, 0.63 | 0.47 | 0.39, 0.57 | 0.02 |
| LysoPC a C20:3 | 2.17 | 1.57, 2.91 | 2.90 | 2.34, 3.59 | <0.0001* | PC ae C30:1 | 0.19 | 0.14, 0.24 | 0.17 | 0.11, 0.25 | 0.38 |
| LysoPC a C20:4 | 9.54 | 7.50, 11.90 | 12.98 | 9.64, 17.07 | <0.0001* | PC ae C30:2 | 0.06 | 0.05, 0.08 | 0.06 | 0.05, 0.08 | 0.70 |
| LysoPC a C24:0 | 0.41 | 0.34, 0.50 | 0.40 | 0.35, 0.47 | 0.07 | PC ae C32:1 | 2.27 | 1.85, 2.94 | 2.13 | 1.80, 2.72 | 0.13 |
| LysoPC a C26:0 | 0.55 | 0.43, 0.69 | 0.46 | 0.37, 0.61 | 0.0004* | PC ae C32:2 | 0.44 | 0.37, 0.52 | 0.41 | 0.35, 0.50 | 0.06 |
| LysoPC a C26:1 | 0.30 | 0.25, 0.37 | 0.27 | 0.23, 0.34 | 0.02 | PC ae C34:0 | 0.86 | 0.69, 1.12 | 0.76 | 0.65, 0.92 | 0.001* |
| LysoPC a C28:0 | 0.44 | 0.36, 0.54 | 0.44 | 0.38, 0.51 | 0.87 | PC ae C34:1 | 6.26 | 4.84, 8.50 | 5.15 | 4.21, 6.42 | <0.0001* |
| LysoPC a C28:1 | 0.48 | 0.40, 0.55 | 0.48 | 0.41, 0.56 | 0.57 | PC ae C34:2 | 5.19 | 4.28, 6.24 | 4.86 | 4.16, 5.63 | 0.04 |
| PC aa C24:0 | 0.19 | 0.16, 0.24 | 0.18 | 0.15, 0.22 | 0.33 | PC ae C34:3 | 3.74 | 3.09, 4.74 | 4.27 | 3.61, 5.10 | 0.002* |
| PC aa C28:1 | 1.05 | 0.82, 1.30 | 1.26 | 0.95, 2.98 | <0.0001* | PC ae C36:0 | 0.55 | 0.45, 0.65 | 0.55 | 0.45, 0.65 | 0.84 |
| PC aa C30:0 | 2.38 | 1.82, 3.20 | 2.48 | 1.77, 3.86 | 0.34 | PC ae C36:1 | 3.87 | 3.00, 5.16 | 3.40 | 2.85, 3.92 | 0.0003* |
| PC aa C30:2 | 0.40 | 0.30, 0.52 | 0.40 | 0.31, 0.55 | 0.39 | PC ae C36:2 | 6.48 | 5.03, 8.16 | 5.42 | 4.53, 6.72 | 0.0001* |
| PC aa C32:0 | 13.7 | 10.6, 17.5 | 11.7 | 9.9, 14.2 | 0.0002* | PC ae C36:3 | 3.49 | 2.91, 4.15 | 3.34 | 2.85, 3.92 | 0.25 |
| PC aa C32:1 | 9.60 | 5.86, 14.26 | 6.42 | 4.27, 9.35 | <0.0001* | PC ae C36:4 | 11.5 | 9.8, 14.5 | 11.9 | 9.9, 15.3 | 0.28 |
| PC aa C32:2 | 1.75 | 1.31, 2.22 | 1.83 | 1.24, 2.62 | 0.29 | PC ae C36:5 | 9.31 | 7.57, 11.10 | 10.68 | 8.41, 14.21 | <0.0001* |
| PC aa C32:3 | 0.24 | 0.20, 0.27 | 0.24 | 0.21, 0.28 | 0.30 | PC ae C38:0 | 0.97 | 0.82, 1.24 | 1.06 | 0.89, 1.23 | 0.08 |
| PC aa C34:1 | 132.6 | 103.6, 165.5 | 105.4 | 84.6, 139.2 | <0.0001* | PC ae C38:1 | 0.39 | 0.29, 0.50 | 0.33 | 0.23, 0.43 | 0.006* |
| PC aa C34:2 | 210.6 | 178.9, 237.4 | 188.3 | 162.3, 216.7 | 0.0008* | PC ae C38:2 | 1.39 | 1.07, 1.78 | 1.15 | 0.96, 1.53 | 0.0009* |
| PC aa C34:3 | 6.47 | 4.88, 8.56 | 4.78 | 3.57, 6.72 | <0.0001* | PC ae C38:3 | 2.10 | 1.65, 2.60 | 1.97 | 1.68, 2.39 | 0.26 |
| PC aa C34:4 | 0.78 | 0.62, 1.00 | 0.95 | 0.72, 1.29 | <0.0001* | PC ae C38:4 | 9.43 | 8.08, 10.95 | 9.10 | 7.97, 11.16 | 0.56 |
| PC aa C36:0 | 1.80 | 1.40, 2.30 | 1.86 | 1.36, 2.63 | 0.20 | PC ae C38:5 | 13.2 | 11.3, 15.9 | 13.1 | 10.8, 15.8 | 0.77 |
| PC aa C36:1 | 29.2 | 23.8, 37.6 | 25.9 | 22.1, 32.8 | 0.005* | PC ae C38:6 | 4.39 | 3.70, 5.42 | 4.93 | 3.96, 6.48 | 0.002* |
| PC aa C36:2 | 130.0 | 111.9, 149.0 | 122.6 | 103.9, 139.4 | 0.03 | PC ae C40:1 | 0.70 | 0.54, 0.87 | 0.73 | 0.55, 0.88 | 0.24 |
| PC aa C36:3 | 66.7 | 52.8, 78.8 | 65.5 | 53.9, 73.4 | 0.76 | PC ae C40:2 | 0.67 | 0.55, 0.81 | 0.65 | 0.54, 0.82 | 0.48 |
| PC aa C36:4 | 134.8 | 110.7, 152.3 | 135.7 | 115.6, 152.2 | 0.46 | PC ae C40:3 | 0.62 | 0.51, 0.75 | 0.61 | 0.52, 0.71 | 0.39 |
| PC aa C36:5 | 6.23 | 4.65, 8.48 | 5.87 | 4.65, 8.03 | 0.65 | PC ae C40:4 | 1.83 | 1.56, 2.09 | 1.75 | 1.54, 2.08 | 0.54 |
| PC aa C36:6 | 0.45 | 0.36, 0.58 | 0.58 | 0.45, 0.77 | <0.0001* | PC ae C40:5 | 2.51 | 2.15, 2.90 | 2.37 | 2.08, 2.75 | 0.09 |
| PC aa C38:0 | 2.30 | 1.89, 2.99 | 2.37 | 1.82, 3.03 | 0.65 | PC ae C40:6 | 3.38 | 2.85, 3.93 | 3.47 | 2.94, 4.30 | 0.25 |
| PC aa C38:1 | 0.74 | 0.49, 1.00 | 0.67 | 0.49, 0.97 | 0.29 | PC ae C42:1 | 0.61 | 0.50, 0.74 | 0.54 | 0.42, 0.69 | 0.001* |
| PC aa C38:3 | 33.4 | 27.5, 41.1 | 35.4 | 30.0, 41.2 | 0.08 | PC ae C42:2 | 0.39 | 0.32, 0.50 | 0.38 | 0.28, 0.48 | 0.27 |
| PC aa C38:4 | 97.5 | 76.5, 113.3 | 97.8 | 83.1, 112.6 | 0.29 | PC ae C42:3 | 0.45 | 0.37, 0.55 | 0.45 | 0.38, 0.57 | 0.70 |
| PC aa C38:5 | 31.8 | 27.0, 39.7 | 32.4 | 27.6, 38.4 | 0.87 | PC ae C42:4 | 0.60 | 0.51, 0.70 | 0.61 | 0.51, 0.70 | 0.38 |
| PC aa C38:6 | 52.8 | 41.0, 63.8 | 61.9 | 48.7, 75.8 | <0.0001* | PC ae C42:5 | 1.54 | 1.37, 1.75 | 1.50 | 1.33, 1.75 | 0.62 |
| PC aa C40:2 | 0.30 | 0.24, 0.38 | 0.29 | 0.21, 0.35 | 0.19 | PC ae C44:3 | 0.26 | 0.21, 0.30 | 0.25 | 0.22, 0.29 | 0.72 |
| PC aa C40:3 | 0.53 | 0.43, 0.64 | 0.52 | 0.40, 0.61 | 0.26 | PC ae C44:4 | 0.30 | 0.26, 0.35 | 0.31 | 0.26, 0.35 | 0.20 |
| PC aa C40:4 | 4.43 | 3.50, 5.59 | 4.10 | 3.48, 5.15 | 0.21 | PC ae C44:5 | 1.09 | 0.91, 1.26 | 1.13 | 0.93, 1.31 | 0.29 |
| PC aa C40:5 | 9.59 | 7.90, 11.82 | 9.59 | 7.91, 11.49 | 0.96 | PC ae C44:6 | 1.62 | 1.38, 1.88 | 1.66 | 1.42, 1.95 | 0.36 |
| PC aa C40:6 | 23.4 | 18.2, 29.3 | 26.0 | 22.8, 32.4 | 0.002* |  | | | | | |

^1^Abbreviations for lipid nomenclature are described in the Methods section.

^2^Wilcoxon rank-sum test. IQR, interquartile range.

*Significant at Bonferroni-adjusted p-value of <0.01

**Supplementary Table 4. Spearman correlations of serum amino acids, biogenic amines, and amino acid metabolites with height-for-age Z-score**

| **Analyte** | ***r*** | ***P*** | **Analyte** | ***r*** | ***P*** |
| --- | --- | --- | --- | --- | --- |
| Tryptophan | 0.29 | <0.0001* | Alanine | 0.06 | 0.27 |
| Isoleucine | 0.26 | <0.0001* | Aspartic acid | 0.04 | 0.52 |
| Leucine | 0.34 | <0.0001* | Glutamate | 0.22 | 0.0001* |
| Valine | 0.25 | <0.0001* | Citrulline | 0.16 | 0.004* |
| Methionine | 0.26 | <0.0001* | Ornithine | 0.27 | <0.0001* |
| Histidine | 0.21 | 0.0002* | Alpha aminoadipic acid | -0.05 | 0.40 |
| Phenylalanine | 0.13 | 0.03 | Kynurenine | -0.05 | 0.38 |
| Threonine | 0.32 | <0.0001* | Creatinine | 0.18 | 0.001* |
| Lysine | 0.25 | <0.0001* | Spermine | -0.06 | 0.31 |
| Asparagine | 0.21 | 0.0001* | Putrescine | -0.07 | 0.21 |
| Glutamine | 0.17 | 0.003* | Serotonin | 0.15 | 0.006* |
| Arginine | 0.20 | 0.0003* | Taurine | 0.25 | <0.0001* |
| Glycine | 0.19 | 0.0008* | Total dimethylarginine | -0.02 | 0.76 |
| Proline | 0.01 | 0.99 | Symmetric dimethylarginine | -0.04 | 0.45 |
| Serine | 0.23 | <0.0001* | Asymmetric dimethylarginine | 0.09 | 0.09 |
| Tyrosine | 0.20 | 0.0003* |  | | |

*Significant at Bonferroni-adjusted p-value of <0.01

**Supplementary Table 5. Spearman correlations of serum sphingolipids^1^ and acylcarnitines with height-for-age Z-score, adjusted by age and gender**

| **Analyte** | ***r*** | ***P*** | **Analyte** | ***r*** | ***P*** |
| --- | --- | --- | --- | --- | --- |
| SM (OH) C14:1 | 0.12 | 0.04 | SM C24:1 | 0.07 | 0.22 |
| SM (OH) C16:1 | 0.12 | 0.03 | SM C26:0 | -0.01 | 0.98 |
| SM (OH) C22:1 | 0.09 | 0.08 | SM C26:1 | 0.02 | 0.71 |
| SM (OH) C22:2 | 0.20 | 0.0003* | Carnitine | 0.19 | 0.0004* |
| SM (OH) C24:1 | 0.10 | 0.07 | Acetylcarnitine | 0.08 | 0.14 |
| SM C16:0 | 0.06 | 0.30 | Propionylcarnitine | -0.07 | 0.19 |
| SM C16:1 | 0.13 | 0.02 | Butyrylcarnitine | 0.08 | 0.18 |
| SM C18:0 | 0.10 | 0.09 | Hydroxybutyrylcarnitine | 0.01 | 0.92 |
| SM C18:1 | 0.17 | 0.003* | Hexadecanoylcarnitine | 0.02 | 0.69 |
| SM C20:2 | -0.08 | 0.15 | Octadecenoylcarnitine (C18:1) | 0.02 | 0.72 |
| SM C22:3 | 0.07 | 0.24 | Octadecadienylcarnitine (C18:2) | -0.01 | 0.98 |
| SM C24:0 | 0.09 | 0.11 |  | | |

^1^Abbreviations for lipid nomenclature are described in the Methods section.

*Significant at Bonferroni-adjusted p-value of <0.01

**Supplementary Table 6. Spearman correlations of serum glycerophospholipids^1^ with height-for-age Z-score, adjusted by age and gender**

| **Analyte** | ***r*** | ***P*** | **Analyte** | ***r*** | ***P*** |
| --- | --- | --- | --- | --- | --- |
| LysoPC a C16:0 | 0.21 | 0.0001* | PC aa C42:0 | 0.02 | 0.71 |
| LysoPC a C16:1 | 0.05 | 0.39 | PC aa C42:1 | 0.09 | 0.11 |
| LysoPC a C17:0 | 0.21 | 0.0002* | PC aa C42:2 | -0.01 | 0.85 |
| LysoPC a C18:0 | 0.30 | <0.0001* | PC aa C42:4 | -0.02 | 0.79 |
| LysoPC a C18:1 | 0.15 | 0.007* | PC aa C42:5 | -0.02 | 0.73 |
| LysoPC a C18:2 | 0.18 | 0.001* | PC aa C42:6 | 0.02 | 0.72 |
| LysoPC a C20:3 | 0.21 | 0.0002* | PC ae C30:1 | -0.17 | 0.003* |
| LysoPC a C20:4 | 0.31 | <0.0001* | PC ae C30:2 | 0.03 | 0.60 |
| LysoPC a C24:0 | 0.08 | 0.18 | PC ae C32:1 | -0.12 | 0.03 |
| LysoPC a C26:0 | -0.18 | 0.0009* | PC ae C32:2 | -0.10 | 0.09 |
| LysoPC a C26:1 | -0.10 | 0.07 | PC ae C34:0 | -0.25 | 0.0001* |
| LysoPC a C28:0 | -0.08 | 0.18 | PC ae C34:1 | -0.29 | <0.0001* |
| LysoPC a C28:1 | 0.02 | 0.73 | PC ae C34:2 | -0.13 | 0.02 |
| PC aa C24:0 | -0.04 | 0.53 | PC ae C34:3 | 0.23 | <0.0001* |
| PC aa C28:1 | 0.12 | 0.04 | PC ae C36:0 | -0.07 | 0.24 |
| PC aa C30:0 | -0.17 | 0.003* | PC ae C36:1 | -0.21 | 0.0002* |
| PC aa C30:2 | 0.05 | 0.34 | PC ae C36:2 | -0.18 | 0.001* |
| PC aa C32:0 | -0.28 | <0.0001* | PC ae C36:3 | -0.08 | 0.15 |
| PC aa C32:1 | -0.31 | <0.0001* | PC ae C36:4 | -0.02 | 0.67 |
| PC aa C32:2 | -0.11 | 0.06 | PC ae C36:5 | 0.17 | 0.002* |
| PC aa C32:3 | 0.03 | 0.59 | PC ae C38:0 | 0.18 | 0.002* |
| PC aa C34:1 | -0.27 | <0.0001* | PC ae C38:1 | -0.13 | 0.02 |
| PC aa C34:2 | -0.21 | 0.0001* | PC ae C38:2 | -0.14 | 0.01* |
| PC aa C34:3 | -0.26 | <0.0001* | PC ae C38:3 | -0.05 | 0.43 |
| PC aa C34:4 | 0.11 | 0.05 | PC ae C38:4 | -0.05 | 0.38 |
| PC aa C36:0 | 0.06 | 0.32 | PC ae C38:5 | -0.04 | 0.49 |
| PC aa C36:1 | -0.16 | 0.004* | PC ae C38:6 | 0.15 | 0.008* |
| PC aa C36:2 | -0.12 | 0.04 | PC ae C40:1 | 0.15 | 0.007* |
| PC aa C36:3 | -0.05 | 0.39 | PC ae C40:2 | 0.01 | 0.93 |
| PC aa C36:4 | 0.03 | 0.60 | PC ae C40:3 | -0.01 | 0.95 |
| PC aa C36:5 | -0.01 | 0.89 | PC ae C40:4 | -0.02 | 0.71 |
| PC aa C36:6 | 0.24 | <0.0001* | PC ae C40:5 | -0.06 | 0.28 |
| PC aa C38:0 | 0.04 | 0.44 | PC ae C40:6 | 0.10 | 0.07 |
| PC aa C38:1 | 0.01 | 0.93 | PC ae C42:1 | -0.08 | 0.17 |
| PC aa C38:3 | 0.09 | 0.12 | PC ae C42:2 | 0.07 | 0.23 |
| PC aa C38:4 | 0.13 | 0.02 | PC ae C42:3 | 0.03 | 0.58 |
| PC aa C38:5 | 0.05 | 0.39 | PC ae C42:4 | -0.06 | 0.28 |
| PC aa C38:6 | 0.21 | 0.0002* | PC ae C42:5 | -0.01 | 0.90 |
| PC aa C40:2 | -0.03 | 0.59 | PC ae C44:3 | -0.01 | 0.89 |
| PC aa C40:3 | -0.01 | 0.98 | PC ae C44:4 | 0.07 | 0.06 |
| PC aa C40:4 | -0.02 | 0.78 | PC ae C44:5 | 0.08 | 0.23 |
| PC aa C40:5 | 0.05 | 0.42 | PC ae C44:6 | 0.03 | 0.55 |
| PC aa C40:6 | 0.22 | <0.0001* |  | | |

^1^Abbreviations for lipid nomenclature are described in the Methods section.

*Significant at Bonferroni-adjusted p-value of <0.01

**SUPPLEMENTARY FIGURE LEGENDS**

**Supplementary Figure 1.** Heat map showing the relationship of serum amino acids, biogenic amines, amino acid metabolites, acylcarnitines, and sphingolipids by HAZ. HAZ is divided into deciles. Abbreviations for lipid nomenclature are described in the methods section. Standard three-letter abbreviations are used for amino acids. Other abbreviations: carnitine (C0), acetylcarnitine (C2), proprionylcarnitine (C3), butyrylcarnitine (C4), hydroxybutyrylcarnitine (C3-DC/C4-OH), hexadecanoylcarnitine (C16), octadecenoylcarnitine (C18:1), octadecadienylcarnitine (C18:2), asymmetric dimethylarginine (ADMA), symmetric dimethylarginine (SDMA), total dimethylarginine (total DMA), alpha-AAA (alpha-aminoadipic acid).

**Supplementary Figure 2.** Heat map showing the relationship of serum glycerophospholipids with HAZ. HAZ is divided into deciles. Abbreviations for lipid nomenclature and other metabolites are described in the methods section.
